# Alcohol-Induced Retrograde Facilitation? Mixed Evidence in a Preregistered Replication and Encoding-Maintenance-Retrieval Analysis

**DOI:** 10.1027/1618-3169/a000569

**Published:** 2023-02-21

**Authors:** J. Quevedo Pütter, E. Erdfelder

**Affiliations:** ^1^ Department of Psychology, School of Social Sciences, University of Mannheim, Mannheim, Germany

**Keywords:** retrograde facilitation, alcohol, interference hypothesis, consolidation hypothesis, conceptual replication, multinomial processing tree (MPT) modeling

## Abstract

**Abstract.** Somewhat counterintuitively, alcohol consumption following learning of new information has been shown to enhance performance on a delayed subsequent memory test. This phenomenon has become known as the retrograde facilitation effect (Parker et al., 1981). Although conceptually replicated repeatedly, serious methodological problems are associated with most previous demonstrations of retrograde facilitation. Moreover, two potential explanations have been proposed, the interference and the consolidation hypothesis. So far, empirical evidence for and against both hypotheses is inconclusive (Wixted, 2004). To scrutinize the existence of the effect, we conducted a pre-registered replication that avoided common methodological pitfalls. In addition, we used Küpper-Tetzel and Erdfelder’s (2012) multinomial processing tree (MPT) model to disentangle encoding, maintenance, and retrieval contributions to memory performance. With a total sample size of *N* = 93, we found no evidence for retrograde facilitation in overall cued or free recall of previously presented word pairs. In line with this, MPT analyses also showed no reliable difference in maintenance probabilities. However, MPT analyses revealed a robust alcohol advantage in retrieval. We conclude that alcohol-induced retrograde facilitation might exist and be driven by an underlying retrieval benefit. Future research is needed to investigate potential moderators and mediators of the effect explicitly.


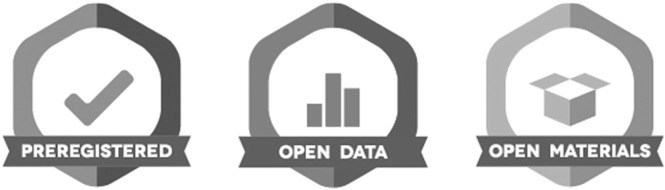


Alcohol is one of the most widely used and abused substances worldwide, with estimated prevalences of current drinking and heavy episodic drinking as high as 47% and 20%, respectively (Manthey et al., 2019). To better understand the consequences of drinking alcohol on everyday functioning, considerable effort has been devoted toward investigating cognitive alterations following acute alcohol intake (Mintzer, 2007). One particularly surprising observation originating from this research was that alcohol consumption following learning of new information apparently enhances performance on a delayed subsequent memory test. Parker et al. (1980) were the first to empirically demonstrate this so-called retrograde facilitation effect in a laboratory experiment using human subjects. While the effect seems to replicate across a variety of experimental paradigms, there are important methodological weaknesses associated with most of the studies used so far. Moreover, the question of which mechanisms underlie retrograde facilitation has rarely been addressed empirically, and the corresponding research has not resulted in conclusive results so far (Wixted, 2004). Both issues are intertwined and of great importance for both theoretical and practical reasons. Therefore, in this registered report, we proposed a replication study that addresses them jointly. Specifically, we suggested a study design that avoids common methodological pitfalls by conceptually replicating the original procedure described by Parker et al. (1981) and, in addition, reanalyzing the replication data using a multinomial processing tree (MPT) model proposed by Küpper-Tetzel and Erdfelder (2012) to disentangle encoding, maintenance, and retrieval contributions to memory performance (for a review of MPT models, see Erdfelder et al., 2009; for an MPT tutorial, see Schmidt et al., 2023). Thus, we addressed two main goals in the present study: (a) rigorous conceptual replication of the retrograde facilitation effect to ensure that it is not an artifact of confounding variables and (b) using an established MPT measurement model to empirically assess the hypotheses proposed to explain retrograde facilitation. The preregistered study protocol is publicly available on the Open Science Framework (OSF; https://doi.org/10.17605/OSF.IO/DK8FJ; Quevedo Pütter et al., 2020).

## Prior Research on Retrograde Facilitation

In 1981, Parker and colleagues demonstrated retrograde facilitation in a highly rigorous manner. In their experiment, alcohol versus placebo administration after learning was manipulated as a within-subjects factor. The retention interval lasted 7 hrs in each case, during which participants remained in a controlled laboratory environment, abstaining from any cognitively challenging tasks and further alcohol consumption. Participants showed significantly better performances in a recognition task after consuming either 0.5 or 1.0 ml alcohol per kg body weight (ml/kg) than after consuming 0.25 ml/kg or no alcohol at all. To illustrate, assuming a body weight of 80 kg, an alcohol weight of 0.8 g/ml, and an alcohol content of 5% for beer, the dose of 0.25 ml/kg would correspond to 20 ml (16 g) of pure alcohol or 0.4 l of beer, the dose of 0.5 ml/kg to 40 ml (32 g) of pure alcohol or 0.8 l of beer, and the dose of 1.0 ml/kg to 80 ml (64 g) of pure alcohol or 1.6 l of beer. Note that these quantities vary as a function of the body weights of individual participants and are thus examples only. Also note that beer is only used as a commonly known reference standard here. Parker et al. (1981) actually used a mixture of pure alcohol and a masking solution in their experiment.

In terms of internal validity, the Parker et al. (1981) study can be considered a convincing test of retrograde facilitation by alcohol. Most importantly, the retention interval of 7 h within a single day is (a) long enough to ensure that participants are completely sober when memory is tested and (b) short enough so that the interval does not cover a night of sleep. Unfortunately, other researchers have tried to avoid long retention intervals in controlled environments by either employing considerably shorter retention intervals (Knowles & Duka, 2004; R. E. Mann et al., 1984; Parker et al., 1980, study 1; Tyson & Schirmuly, 1994) or by dismissing participants after the learning phase and extending the retention interval up to 48 h (Bruce & Pihl, 1997; Carlyle et al., 2017; Lamberty et al., 1990; Mueller et al., 1983; Parker et al., 1980, study 2; Weafer et al., 2016a, 2016b). As detailed below, both approaches have considerable drawbacks compared to the original procedure used by Parker et al. (1981).

Short retention intervals involve the risk that participants are still intoxicated when memory is tested. To illustrate, R. E. Mann et al. (1984) conducted two separate studies with retention intervals of approximately 80 min and 140 min. They reported that participants were intoxicated to a considerable degree when memory was tested and rightfully concluded that memory tests conducted in a sober state for both the placebo and alcohol groups could have led to different results. One obvious criticism is that memory performance in the alcohol condition is hampered under such circumstances because of state-dependent learning (Goodwin et al., 1969). Accordingly, memory performance of participants in the alcohol condition probably suffered from discrepant internal states during learning (sober state) and testing (intoxicated state). Nonetheless, statistically significant retrograde facilitation effects emerged in both studies of R. E. Mann et al. (1984). In principle, this finding is in line with a strong and robust retrograde facilitation effect. However, as participants in the alcohol condition were sober during learning and intoxicated during testing, the true size of the effect is probably underestimated, making it difficult to infer anything about its practical relevance. Even more importantly, as detailed below, one potential explanation of retrograde facilitation by alcohol – the interference hypothesis – can only be tested in a strict manner if accessibility of information in memory is not hampered by unrelated factors such as the state-dependency of memory.

An alternative approach to avoid long retention intervals in supervised controlled environments involves use of retention intervals extending to the next day. Hence, learning and memory testing take place on subsequent days, and participants can be sent home after the first session. This procedure has been employed by various researchers, with retention intervals ranging between 16 hrs (Carlyle et al., 2017) and 48 hrs (Weafer et al., 2016a, 2016b), always encompassing at least 1 night of sleep. While this approach avoids the problem of ongoing alcohol intoxication during recall, it comes with two other, possibly even more severe methodological challenges.

First, sleep research has shown that a presleep dose of alcohol typically increases slow-wave sleep (SWS) in the first half of the night for both healthy adults (Ebrahim et al., 2013) and adolescents (Chan et al., 2013). This poses serious problems for retrograde facilitation research because sleep, and especially SWS, has been argued to play a predominant role in the consolidation of declarative memories (Rasch & Born, 2013). Accordingly, it is possible that the apparent effects of retrograde facilitation by alcohol in these studies are actually due to memory consolidation during sleep. Alternatively, it could also be argued that effects of alcohol on sleep architecture may lead to a decline in memory performance. For example, an increase in sleep disruption following a presleep dose of alcohol (Ebrahim et al., 2013) could deteriorate participants’ test performance on the following day due to sleep deprivation and increased daytime sleepiness during testing. However, this objection was taken into account in two studies by Weafer et al. (2016a, 2016b) who used a retention interval of 48 h to rule out that participants perform memory tests in a sleep-deprived or hungover state.

Second, acute alcohol intake has been shown to interact with sleep deprivation to increase daytime sleepiness (Roehrs & Roth, 2001). Such an effect may be linked to the first problem, as daytime napping during the retention interval has been found to increase declarative memory performance (Tucker et al., 2006).

## Mechanisms Proposed to Underlie Retrograde Facilitation by Alcohol

Two explanations for retrograde facilitation by alcohol have been proposed, the interference hypothesis and the consolidation hypothesis (Mueller et al., 1983; Tyson & Schirmuly, 1994). According to the interference hypothesis, retrograde facilitation is due to new incoming information being encoded and stored in memory less efficiently during intoxication, resulting in anterograde amnesia or at least mild forms thereof. As a consequence, memory representations originating from the previous learning phase are protected from retroactive interference. It has been shown convincingly that deterioration of memory following similarity-based retroactive interference is not due to distortion or loss of memory representations, but rather caused by impaired accessibility of information available in memory (e.g., Lohnas et al., 2015; Tulving & Psotka, 1971). By implication, the interference hypothesis predicts that retrograde facilitation results from enhanced accessibility of learned information in the testing phase, not from improved maintenance across the retention interval. In contrast, proponents of the consolidation hypothesis argue that alcohol actively boosts postlearning processing, resulting in more stable and durable memory representations (Parker et al., 1981), for example, by acting on brain regions involved in learning and memory such as the hippocampus (White, 1996) or by providing ideal (i.e., interference-free) conditions for undisrupted memory consolidation (Mednick et al., 2011; Wixted, 2004, 2010). Hence, retrograde facilitation emerges as a consequence of enhanced maintenance across the retention interval. In sum, while the interference hypothesis posits that alcohol-induced retrograde facilitation effects are due to better retrieval as a consequence of reduced retroactive interference, the consolidation hypothesis identifies better maintenance in memory as the main cause.

Both hypotheses have received some empirical support so far. Specifically, the interference hypothesis is in line with the findings by Mueller et al. (1983) and Tyson and Schirmuly (1994), respectively, showing that (a) retrograde facilitation is not time-dependent so that its strength does not depend on whether alcohol administration directly follows the learning phase or occurs later (although it has been shown that retroactive interference is also time-dependent to a certain degree; for a review, see Wixted, 2004) and (b) that retrograde facilitation is more pronounced in memory tasks with few or no retrieval cues (e.g., free recall tasks) compared to tasks providing strong retrieval cues (e.g., recognition tasks), that is, effect sizes between alcohol and placebo conditions are more pronounced in free recall tasks than in recognition tasks. In contrast, Parker et al. (1981) favor a consolidation explanation because they observed a retrograde facilitation effect although a relatively low dose of alcohol was used in one condition (0.5 ml/kg) that is unlikely to decrease retroactive interference significantly. Perhaps more importantly, cognitive activity during the retention interval was reduced to a minimum in their study, making protection against interfering information obsolete.

## Measurement of Memory Maintenance and Retrieval Contributions

Based on the evidence summarized in the previous section, Wixted (2004) concluded that attempts to empirically discriminate between the interference and the consolidation hypothesis have been inconclusive so far. He supposed that identification of the mediating physiological mechanisms might be a precondition for discriminating between the two theoretical accounts successfully. Following a different rationale, we propose an appropriate MPT model (see Erdfelder et al., 2009, for a review) to disentangle maintenance and retrieval contributions to the retrograde facilitation effect on a functional rather than a physiological level.

One particularly promising MPT model for our purposes is the encoding-maintenance-retrieval (EMR) model (Küpper-Tetzel & Erdfelder, 2012), an extension of the previously proposed storage-retrieval model by Rouder and Batchelder (1998). Küpper-Tetzel and Erdfelder’s EMR model measures encoding, maintenance, and retrieval contributions to overall memory performance. It requires a study design that involves study of paired associates, immediately followed by a cued recall task. After a retention interval, additional free and final cued recall tests are administered (free-then-cued-recall paradigm). As detailed below, these methodological requirements are easily combined with the typical retrograde facilitation research design, enabling us to decide empirically whether retrograde facilitation by alcohol – if it exists – is driven by (a) improved maintenance across the retention interval, (b) improved retrieval in delayed subsequent free recall, or (c) both. Crucially, as shown above, the interference hypothesis and the consolidation hypothesis clearly differ with respect to which of these processes are expected to underlie retrograde facilitation. Also, because these hypotheses are not mutually exclusive, it is conceivable that both are correct, as reflected in improvements of both maintenance and retrieval after alcohol consumption.

Because the EMR model relies on a study design encompassing one initial cued recall test (2 possible outcomes per studied word pair: correct vs. incorrect), one later free recall test (3 possible outcomes: 0, 1, or 2 words of a pair recalled), and one final cued recall test (2 possible outcomes: correct vs. incorrect), a total of 2 × 3 × 2 = 12 observable outcome patterns E_1_ to E_12_ can occur for each word pair, depending on participants’ performance in the three memory tests (cf. Table 1). The model comprises seven latent parameters: the probability of successful encoding of an association (*e*), the probabilities of maintenance of stored associations across the retention interval given successful versus unsuccessful initial cued recall (*m*_s_ and *m*_u_, respectively), the probabilities of successful retrieval in free (*r*_f_) and cued recall (*r*_c_), and finally, the probabilities of single word retrieval in free recall given successful versus unsuccessful associative encoding (*s* and *u*, respectively). In total, the model includes 32 possible sequences of successful versus unsuccessful encoding, maintenance, and retrieval steps (so-called branches), each terminating in one of the 12 event categories E_1_ to E_12_. The probability of a branch is just the product of parameters along that branch, and the probability of a category equals the sum of the branch probabilities corresponding to this category. Based on these rules, model equations are obtained that represent the probabilities of the observable categories E_1_ to E_12_ as functions of the seven model parameters. Given these equations and a set of event frequencies, model parameters can be estimated using either standard maximum likelihood techniques (Batchelder & Riefer, 1999; Hu & Batchelder, 1994; Moshagen, 2010) or Bayesian estimation techniques for hierarchical model versions that account for individual differences in model parameters (Heck et al., 2018). Küpper-Tetzel and Erdfelder (2012) have previously tested and validated the model successfully. Both an illustration of the model and the 12 model equations are available in the Electronic Supplemental Material (ESM) 1.

**Table 1:** Twelve observable event categories E_1_ to E_12_ for a study design employing an initial cued recall immediately following the learning phase and a free-then-cued-recall in the recall phase

Initial cued recall	Final cued recall	Final free recall		
		Both words	Exactly one word	Neither word
Correct	Correct	E_1_	E_2_	E_3_
	Incorrect	E_4_	E_5_	E_6_
Incorrect	Correct	E_7_	E_8_	E_9_
	Incorrect	E_10_	E_11_	E_12_
*Note*. Twelve possible memory test outcomes in the 3-level free-then-cued-recall paradigm proposed by Küpper-Tetzel and Erdfelder (2012).				

For our purposes, maintenance (*m*) and retrieval parameters (*r*_f_) are key parameters to test the consolidation and the interference hypothesis, respectively. As detailed above, the interference hypothesis predicts that retrograde facilitation results from enhanced accessibility of learned information in the testing phase. In the EMR model, this process is represented by the retrieval parameter *r*_f_, the probability of successful retrieval of stored associations in free recall. In contrast, parameter *r*_c_ represents the probability of successful retrieval of the second word when the first word of an association stored in memory is provided as a cue. Given that the word pair is stored in memory, this cued recall probability should generally be very close to 1 in all conditions, as was previously found by Küpper-Tetzel and Erdfelder (2012). Parameter *r*_c_ is thus irrelevant for testing our hypotheses.

The consolidation hypothesis predicts that retrograde facilitation emerges as a consequence of enhanced maintenance across the retention interval. This process is represented by the maintenance parameters *m*_s_ and *m*_u_. Küpper-Tetzel and Erdfelder (2012) observed that *m*_s_ and *m*_u_ can be equated to a single maintenance parameter *m*. Accordingly, the consolidation hypothesis predicts that *m* is larger in the alcohol condition compared to the placebo condition.

Parameter *e* should not be affected by alcohol administration after learning as it represents successful encoding of an association, a process that takes place before the experimental manipulation. Finally, parameters *s* and *u* represent probabilities of successful single word maintenance and retrieval for associations stored versus not stored in memory, respectively. They are thus not informative with respect to the question whether alcohol specifically enhances associative maintenance or retrieval.

It is important that the two parameters of prime interest, *m* and *r*_f_, are not influenced by factors other than the administration of alcohol versus a placebo. It thus needs to be assured that (a) participants are completely sober when memory is tested after the retention interval to avoid contaminations of *r*_f_ with state-dependent learning decrements and (b) the retention interval does not include a night of sleep or daytime naps to preclude contaminations of successful maintenance (*m*) and retrieval (*r*_f_) with sleep-related differences between conditions. For this purpose, we largely followed the original study by Parker et al. (1981) and employed a retention interval of 7 h that ensured a sober state at both study and test. Additionally, the procedure was conducted in the controlled environment of a laboratory to preclude sleep, further consumption of alcohol, and other confounding variables. Note, however, that several methodological differences between our study and the original Parker et al. (1981) study remain. First, we did not restrict cognitive activity during the retention interval in any way (apart from enforcing compliance with the instructions). According to the interference hypothesis, the more cognitive activity during retention, the more accessibility benefits should emerge in the alcohol condition. Thus, a fair test of the interference hypothesis requires considerable cognitive activity in both conditions. Second, we used cued and free recall tasks for paired associates rather than assessing recognition memory for pictures. This adjustment is required by the EMR model. The same holds for the initial cued recall test immediately following learning, a procedure that deviates from Parker et al. (1981) but is mandatory for the EMR model-based analysis. Third, alcohol consumption was manipulated as a between-subjects factor in our study. We feel that this prevents alcohol-related expectancy effects more effectively than a within-subjects manipulation because participants lack a direct reference standard for the alcohol content. Fourth, we did not restrict our study to male participants but investigated all genders. We took into account the difference in alcohol tolerance between genders by administrating different doses to women (0.51 g/kg) and men (0.59 g/kg).

## Hypotheses

Because the retrograde facilitation effect emerged in all prior studies we were aware of, even in those in which its strength was probably hampered by state-dependent learning influences, we assumed that the effect truly exists and thus hypothesized that forgetting in cued recall across the retention interval (i.e., the difference in correct responses between the initial and the final cued recall) would be reduced for participants in the alcohol condition compared to the placebo condition (Hypothesis 1) and, in addition, that participants in the alcohol condition would retrieve more word associations in final free recall (Hypothesis 2).

Moreover, in line with the interference hypothesis, we hypothesized that the EMR model probability of retrieving word associations in free recall (parameter *r*_f_) would be significantly higher for participants in the alcohol condition (Hypothesis 3). Based on the consolidation hypothesis, we additionally hypothesized that maintenance of stored word associations across the retention interval (parameter *m*) would be significantly higher for participants in the alcohol condition (Hypothesis 4). No specific predictions applied to other model parameters.

## Method

All methodological details of the present study were planned and evaluated by the authors in accordance with the ethical principles outlined in the Declaration of Helsinki (2013). The research protocol was approved by the ethics committee of the University of Mannheim. The materials necessary to replicate this study are available on the OSF (https://doi.org/10.17605/OSF.IO/8E9PW).

### Design

The present study was conducted in a double-blind, randomized, placebo-controlled manner. Alcohol administration was a between-subjects factor. Thus, one group of participants consumed an individually determined dose of alcohol after learning while another group of participants received a perceptually indistinguishable placebo beverage. Participants were randomly assigned to one of the two conditions.

### Participants

#### Sample Size

We defined a medium effect size of *d* = 0.50 as the minimum effect of interest in the present study. With *d* = 0.50, α = .05, and a desired statistical power of 1 − β = .80, a conventional Neyman-Pearson power analysis using the software G*Power (Faul et al., 2007) results in a required sample size of *N* = 102 for a one-tailed (i.e., directed) 2-groups *t* test (51 per experimental condition). To maximize efficiency, a sequential *t* test (e.g., Schnuerch & Erdfelder, 2020) was used to test the differences in cued and free recall memory performance between conditions, an approach that has been shown to reduce the required sample size to about 60% of the corresponding Neyman-Pearson sample size on average (see below). If both sequential tests would terminate with *n* < 30 in either condition, additional participants would be sampled subsequently until the threshold *n* = 30 would have been reached in each condition to enable meaningful MPT analyses of the data.

#### Recruitment

Potential participants were recruited via the online platform for study participation of the University of Mannheim and by advertisement within the university campus. Participants received study credit. The top three performances in the immediate cued recall and the final free-then-cued-recall were rewarded with 20 € each. Study information advised subjects to participate in groups because they would watch movies, eat pizza, and drink alcohol as part of a study taking 8.5 hrs in total and allegedly investigating processing of movies under the influence of alcohol.

#### Eligibility

Interested individuals were required to report at least one heavy drinking episode (five alcoholic beverages for men and four alcoholic beverages for women at one occasion; Wechsler et al., 1995) within the last month to ensure that they had sufficient experience to tolerate the to-be-consumed alcohol dose. Additional inclusion criteria included age between 18 and 29 years, body mass index (BMI) between 18.5 and 29.9, no current or past diagnosis of a substance use disorder or any other addictive disorder, no current diagnosis of any other psychiatric disorder, no physical disorder that precludes the consumption of alcohol, no medical advice to avoid the consumption of alcohol, no current intake of any medication other than birth control, no pregnancy or possibility of pregnancy, and no lactation. Age and BMI restrictions were included to allow precise estimation of blood alcohol concentrations (BACs) by the Widmark formula (Thierauf et al., 2013). All criteria were assessed by self-report. Social drinking and no substance use disorder were further confirmed through the Alcohol Use Disorders Identification Test (AUDIT; see below).

### Materials

#### Alcohol and Placebo

For participants assigned to the alcohol condition, the alcohol dose was 0.51 g/kg for women and 0.59 g/kg for men.^1^ Based on the Widmark formula (Widmark, 1932, as cited in Thierauf et al., 2013) and following the refined procedure outlined by Thierauf et al. (2013), these doses could be expected to result in BACs of around 0.60‰ (see ESM 2), an intoxication level high enough to cause significant cognitive impairment. Assuming an elimination rate of around 0.15‰ per hour (Thierauf et al., 2013), it could be expected that alcohol would be eliminated from the body within the retention interval of 7 h. Moreover, the doses lie within the range of dosages that have successfully been employed in previous research on retrograde facilitation, ranging from 0.40 g/kg (Parker et al. 1981) to 0.80 g/kg (e.g., Weafer et al., 2016a). The difference between the two doses for women and men accounts for the fact that women typically exhibit higher BACs than men for a given amount of consumed alcohol (Fillmore, 2001).

In the alcohol condition, a mixture of vodka, tonic water, and Tabasco sauce was served as described by Knowles and Duka (2004). The beverage in the alcohol condition was made up of one part vodka (40% alcohol content) and three parts tonic water such that the total amount of alcohol reached the individually predetermined dose. For administration, each individual beverage was split into 10 portions, meaning that every participant was asked to consume their respective total beverage in 10 smaller portions. To mask the taste and burn of vodka, two drops of Tabasco sauce were added to each portion. In the placebo condition, vodka was replaced with additional tonic water. In both conditions, glass rims were swabbed with vodka to further intensify the sensual impression of alcohol in the placebo condition (Lamberty et al., 1990). Hence, participants in the placebo condition actually did consume some alcohol, but a very low and negligible dose. Due to the inclusion criterion of a BMI between 18.5 and 29.9, the total amount of individual beverages in the alcohol condition could range between 252 ml (67 ml vodka + 185 ml tonic water, split into 10 portions of 25.2 ml each) in case of female gender and body weight of 42 kg, and 832 ml (221 ml vodka + 611 ml tonic water, split into 10 portions of 83.2 ml each) in case of male gender and body weight of 120 kg. Note that these values are theoretical extremes that were not encountered in our study. Actual beverages varied between 266 ml (71 ml vodka + 195 ml tonic water, split into 10 portions of 26.6 ml each) and 624 ml (166 ml vodka + 458 ml tonic water, split into 10 portions of 62.4 ml each).

#### Word Pairs

Forty German word pairs were taken from Hager and Hasselhorn (1994), the same as used by Küpper-Tetzel and Erdfelder (2012). These word pairs have the important characteristic of being only weakly associated to another. Hence, the probability of successful generation of the target word by mere guessing when presented with the cue word in cued recall tasks is minimized. A complete overview of the learning material is provided in ESM 3.

#### AUDIT

A German translation of the Alcohol Use Disorders Identification Test (AUDIT; Saunders et al., 1993) was used to check the exclusion criterion of hazardous drinking habits and the inclusion criterion of social drinking. This scale is recommended in German clinical guidelines for the screening of alcohol use disorders (K. Mann et al., 2017). It consists of 10 items asking for the amount and frequency of drinking occasions and the occurrence of several negative consequences following drinking. Items include three to five alternatives that are rated with a score between 0 and 4. Thus, the maximum total score is 40. Total scores of 8 or more are treated as indicators of harmful alcohol use (Babor et al., 2001). Therefore, interested persons scoring above 7 were not allowed to participate. The inclusion criterion of social drinking was ensured by allowing participation only if individuals reached a total score of at least 2 on the first two items (i.e., one drinking occasion per month with at least three to four alcoholic drinks or two to four drinking occasions per month with at least one or two alcoholic drinks) and a score of at least 1 on the third item (i.e., heavy drinking episodes in the past).

### Procedure

Individuals interested in participating in this study first accessed an online survey that provided them with a description of the study procedure. To prevent demand effects, participants were told that (a) all participants in this study would consume alcohol in a more or less high dose and that (b) the focus of this study lay on the perception and processing of movies under the influence of alcohol. Inclusion criteria were also checked at this occasion. Eligible participants were asked not to drink alcohol within 48 hrs prior to their participation in the experiment and to have lunch before participation began. The experiment took place in a classroom-like laboratory within the premises of the Department of Psychology at the University of Mannheim.

Participants were asked to arrive at 1:30 p.m. at the laboratory. With the exception of general information and instructions regarding the procedure, the first phase of the experiment was administered individually. First, participants were asked for inclusion criteria again and provided written consent. Additionally, breath alcohol concentration was measured to ensure soberness of all participants. Then, participants were instructed for the learning phase. Forty word pairs were presented in randomized order for 5 s each on a computer screen, with participants being informed that memory for the materials would be tested afterward in a cued recall task. The announced immediate cued recall was conducted following a short distractor task where participants were asked to sequentially assess the correctness of 15 equations as quickly as possible. In the immediate cued recall, participants were required to complete each cue word with the respective target word. Cue words were presented in randomized order on a computer screen with participants responding by filling in the respective target words at their own pace. After completion, participants were asked to drink 10 portions of a beverage (either the alcohol or the placebo beverage, depending on the condition they were assigned to) within 30 min (i.e., 3 min per portion). Both the experimenter and the participant were blind with respect to the experimental condition. Following the first portion, participants were asked to estimate the alcohol content of the beverage on a scale from “less than 1%” to “equal to or more than 20%” (note that the true value was about 10% for all participants in the alcohol condition) to check whether the placebo beverage successfully blinded participants for their experimental condition (Keane et al., 1980). After consuming the final portion, participants of both conditions were asked to rinse their mouth with water. Participants were instructed individually not to communicate with other participants about their experiences during learning and alcohol administration. This completed the first phase of this study that lasted approximately 1 h.

The retention interval of 7 h was spent by participants in a seminar room under permanent supervision. Because participants were encouraged to register in groups, they could freely interact and socialize with their colleagues during this time. As part of the cover story, movies were presented. As participation in the study covered the evening hours, pizza was provided for dinner. Additionally, nonalcoholic drinks were available all the time. Breath alcohol concentrations of all participants were checked 30, 60, and 90 min after the administration of alcohol or a placebo (to assess peak BACs) and immediately before memory testing began (to ensure soberness of all participants). Participants were told to abstain from drinking alcohol and sleeping over the whole course of the retention interval. Compliance with the instruction not to communicate about the learning phase and the alcohol administration was monitored by the experimenter.

After the retention interval, participants performed a surprise free-then-cued-recall for the learned word associations. In the free recall task, participants were asked to recall as many of the word pairs as possible within 8 min. Importantly, participants were instructed to write down single words in cases when they did not remember both words of an association. Next, a final cued recall identical to the immediate cued recall task after the study phase was employed. Additionally, participants were asked to declare whether they followed the instruction not to communicate about their experiences from the first phase of the experiment and whether they thought about or actively rehearsed any word pairs in a postexperimental questionnaire. Finally, participants were informed about the true theoretical background and design of the study and the exact dose of alcohol they consumed. Additionally, research assistants were present to answer further questions and concerns. The study ended by 10.00 p.m.

### Data Analysis

All statistical tests were conducted with α = .05. As a manipulation check, peak BACs in the alcohol condition were expected to be significantly larger than zero and significantly larger than BACs observed in the placebo condition. BACs in the placebo condition were expected never to rise to a level larger than zero. Blinding of participants for their condition would be regarded as successful if participants in the placebo condition estimated the alcohol content of their beverage as 1% or more, indicated by all responses other than „less than 1%”. Analyses were conducted with and without those participants in the placebo condition who estimated the alcohol content as less than 1% to test whether results were affected by BAC awareness. Similarly, analyses were conducted with and without those participants who communicated about Phase 1 of the experiment with other participants.

To maximize efficiency of data collection, the sampling process was conducted using a one-tailed group-sequential probability ratio (SPRT) *t* test. Thus, after each group of participants investigated as described above, the resulting data were analyzed to decide whether a decision for or against Hypothesis 1 could be made or further data were required. This sequential procedure has been shown to result in a considerable reduction of required sample sizes when compared to conventional Neyman-Pearson *t* tests. At the same time, error probabilities are controlled (Schnuerch & Erdfelder, 2020). In our application, standard parameters for the Type-1 and Type-2 error probabilities were used (i.e., α = .05 and β = .20, respectively, assuming a medium effect size *d* = .50, cf. Cohen, 1988). Difference scores were calculated for every participant, subtracting the number of correct responses in the final cued recall from the number of correct responses in the immediate cued recall. Then, the difference between the two mean difference scores of experimental conditions were tested for statistical significance using the SPRT *t* test as specified above until a decision was made.

To test Hypothesis 2, the number of complete word pairs recalled was calculated for every participant to obtain mean performances for both experimental conditions. The difference of these two means was tested for statistical significance with the same group-sequential one-tailed two-sample SPRT *t* test as in case of Hypothesis 1.

Hypotheses 3 and 4 referred to parameters *r*_f_ and *m* in the EMR model. Participants underperforming (<30% correct) or overperforming severely (>80% correct) in the immediate cued recall needed to be excluded from MPT analyses to ensure sufficient data points for event categories E_1_ to E_6_ and E_7_ to E_12,_ respectively. A minimum sample size of *n* = 30 per condition was ensured to enable trustworthy parameter estimates for the EMR model in either of the conditions. Next, the frequencies of the 12 event categories E_1_ to E_12_ were calculated individually for all remaining participants and aggregated within conditions. The resulting data could then be used to fit the EMR model (see above). All MPT analyses were conducted twice using TreeBUGS (Heck et al., 2018) for individual data and multiTree (Moshagen, 2010) for aggregated data. Following Küpper-Tetzel and Erdfelder (2012), we would (a) try to equate *m*_s_ and *m*_u_ so that a single parameter *m* represents the probability of successful maintenance and (b) equate *r*_c_ across experimental conditions. For the aggregated data, this leads to 2 (= number of conditions) × 5 (= unrestricted model parameters) + 1 (= number of parameters equated for both conditions) = 11 model parameters to be estimated given 2 (= number of conditions) × 11 (= number of free categories per condition) = 22 independent category frequencies. Accordingly, the number of degrees of freedom of the *G*^2^ goodness‐of‐fit tests test is 22 − 11 = 11. We would accept the model fit if *G*^2^(11) would result in a *p* value greater than .05. If this criterion would not be met, the model would need to be replaced by a more general model version, for example, by allowing *m*, *s*, and/or *u* to differ between successful and unsuccessful immediate cued recall.

To test Hypothesis 3, an equality constraint would be imposed on the *r*_f_ parameters of both experimental conditions. If the resulting decrease in model fit ∆*G*^2^(1) would become significant at α = .05 (one-tailed) and parameter estimates would be in the direction predicted by the interference hypothesis, the latter hypothesis would be confirmed. For the test of Hypothesis 4, analogous procedures would be used: An equality constraint would be imposed on the *m* parameters of both experimental conditions, and the hypothesis would be confirmed if the resulting decrease in model fit ∆*G*^2^(1) would become significant at α = .05 (one-tailed) and parameter estimates would be in the predicted direction.

## Results

The full data set used for the analyses and the corresponding codebook are provided on the OSF (https://doi.org/10.17605/OSF.IO/2K4J7). Additionally, the R code to obtain the results reported below is provided in ESM 4.

### Sample

A total of *N* = 93 participants (divided into 20 sequential groups, 2–10 participants per group, 46 participants in the alcohol condition) took part in the experiment. Although a decision for both SPRT *t* tests was already reached earlier (see below), it was not before this point of the data collection process that the preregistered minimum of *n* = 30 participants per condition was achieved for our encoding-maintenance-retrieval MPT analyses. In the full sample, 62 participants were female, and 31 participants were male. The mean age was 20.67 years (*SD* = 2.18). A majority of 91 participants reported to be university students, of which 72 participants were enrolled in a psychology program. The mean AUDIT score was 5.39 (*SD* = 1.23).

### Control Variables and Manipulation Checks

BAC measurements at the beginning of the experiment confirmed that all participants arrived at the laboratory completely sober. Six participants reported to have consumed alcohol within 48 h before the experiment. Most participants in the alcohol condition (41 of 46 participants) reached their measured peak BAC 30 min after the end of the alcohol administration, four participants reached it after 60 min, and one participant after 90 min. The mean peak BAC in the alcohol condition was 0.43‰ (*SD* = 0.08, Range = 0.22–0.62). At all three measurement occasions after the alcohol administration, mean BACs in the alcohol condition were significantly larger than zero: After 30 min, *M*_BAC_ = 0.43‰ (*SD* = 0.08, Range = 0.22–0.62), *t*(45) = 34.74, *p* < .001; after 60 min, *M*_BAC_ = 0.37‰ (*SD* = 0.08, Range = 0.18–0.57), *t*(45) = 31.40, *p* < .001; and after 90 min, *M*_BAC_ = 0.30‰ (*SD* = 0.08, Range = 0.12–0.46), *t*(42) = 24.91, *p* < .001.^2^ Eight of 47 participants in the placebo condition estimated the alcohol content of their beverage to be below 1%. Actual BACs in the placebo condition never rose to a level larger than zero, except for one participant who had a BAC measure of 0.08‰ 90 min after the end of the placebo administration. All participants in both conditions were completely sober again at the last BAC measurement immediately before the final recall tests.

In the postexperimental questionnaire, 23 participants (13 from the alcohol condition, 10 from the placebo condition) reported to have communicated about their beverages, and two participants (one from the alcohol condition, one from the placebo condition) reported to have communicated about both the beverages and the word pairs during the retention interval. Thirty-three participants (14 from the alcohol condition, 19 from the placebo condition) reported to have thought about the word pairs during the retention interval. Of these, three participants (two from the alcohol condition, one from the placebo condition) reported to have engaged in active rehearsal of the word pairs.

### Design-Based Results

Hypotheses 1 and 2 referred to the difference in correct responses between immediate and final cued recall and the number of complete word pairs reproduced during free recall, respectively. Both hypotheses were tested using the SPRT *t* test (Schnuerch & Erdfelder, 2020) implemented in the sprtt R package (v0.1.0; Steinhilber et al., 2021). For both Hypotheses 1 and 2, the decision to accept the null hypothesis of no significant alcohol benefit was reached rather quickly after *N* = 18 subjects (divided into five sequential groups, *n*_Alcohol_ = *n*_Placebo_ = 9) had participated. The means and standard deviations from this subsample and the full sample (*N* = 93) for the immediate cued recall, the final cued recall, the cued recall difference (Hypothesis 1), the number of complete word pairs reproduced during free recall (Hypothesis 2), and the number of single words reproduced during free recall are reported in Table 2.

**Table 2:** Means and standard deviations (SD) of all dependent variables in the alcohol and placebo condition

Dependent variable	SPRT subsample (*N* = 18)		Full sample (*N* = 93)	
	Alcohol	Placebo	Alcohol	Placebo
Immediate cued recall	19.33 (7.94)	26.56 (4.64)	23.57 (8.89)	23.83 (7.50)
Final cued recall	16.78 (8.80)	24.89 (4.83)	21.85 (9.59)	21.34 (7.65)
Cued recall difference	2.56 (1.81)	1.67 (1.50)	1.72 (1.73)	2.49 (2.41)
Free recall: complete pairs	9.22 (4.60)	13.22 (5.09)	11.57 (5.44)	10.04 (4.66)
Free recall: single words	23.67 (8.89)	31.56 (9.89)	28.11 (10.68)	26.21 (9.29)
*Note*. Decisions in favor of H_0_ for Hypotheses 1 (cued recall difference) and 2 (free recall: complete pairs) were reached by the sequential probability ratio *t* tests (SPRT *t* tests) after data from *N* = 18 participants had been collected. Due to the second stopping criterion of *n* = 30 in both conditions for the multinomial processing tree (MPT) analysis (after excluding participants over- or underperforming in the immediate cued recall), a total of *N* = 93 subjects participated in this study. *SDs* in parentheses.				

For Hypothesis 1, the likelihood ratio (LR) of the SPRT *t* test at *N* = 18 was LR_18_ = 0.18, thereby undercutting the lower SPRT threshold implied by our preregistered SPRT parameters, that is, β/(1 − α) = 0.20/0.95 = 0.21. The observed LR indicates that the data at *N* = 18 were about 1/0.18 = 5.6 times more likely under H_0_ than under H_1_, thus enforcing acceptance of H_0_. The sample estimate of Cohen’s *d* was −0.53, that is, there was a medium-sized effect in the direction opposite to Hypothesis 1. For Hypothesis 2, we observed LR_18_ = 0.11. Thus, at *N* = 18, the data were about 1/0.11 = 9.1 times more likely under H_0_ than under H_1_, also enforcing acceptance of H_0_. In this case, there was a strong effect in the direction opposite to Hypothesis 2, Cohen’s *d* = −0.82. In sum, no significant retrograde facilitation effect could be observed for either of our two dependent variables. Plots of the developments of the log-likelihood ratios for both SPRT *t* tests are provided in ESM 5.

Although irrelevant for the SPRT *t* test decisions in favor of H_0_, we additionally inspected the likelihood ratios of the full-sample SPRT *t* tests, LR_93_, for explorative reasons. Moreover, because LR_93_ assumes a fixed effect size of *d* = 0.50 under H_1_ – a debatable assumption – we additionally computed corresponding Bayes factors (BF_10_) using the default Cauchy prior as implemented in the BayesFactor R package (v0.9.12-4.2; Morey & Rouder, 2018). As summarized in Table 2, the descriptive pattern regarding our two dependent variables of interest was reversed in the full sample of *N* = 93 as compared to the subsample of *N* = 18 required until termination of the SPRT. However, likelihood ratios in the full sample indicate no clear evidence in favor of H_1_ in both cases; LR_93_ = 3.84, Cohen’s *d* = 0.37 for Hypothesis 1 and LR_93_ = 1.81, Cohen’s *d* = 0.30 for Hypothesis 2. In line with this, Bayesian *t* tests revealed BF_10_ = 1.63 for Hypothesis 1 and BF_10_ = 1.00 for Hypothesis 2. Thus, although Hypotheses 1 and 2 are descriptively in line with the data of the full sample, the evidence in favor of H_1_ is negligible in both cases (Kass & Raftery, 1995).

To further scrutinize the robustness of our conclusions regarding Hypotheses 1 and 2, we excluded participants from both the subsample and the full sample who either (a) were assigned to the placebo condition and estimated the alcohol content of their beverage to be less than 1% or (b) reported to have communicated about Phase 1 of the experiment with other participants. The means and standard deviations for the two dependent variables of interest are reported in Table 3.

**Table 3:** Means and standard deviations (SD) of both dependent variables of interest in the alcohol and placebo conditions after exclusion criteria were applied

Dependent variable	SPRT subsample (*N* = 12)		Full sample (*N* = 62)	
	Alcohol	Placebo	Alcohol	Placebo
Cued recall difference	2.67 (2.07)	2.00 (1.26)	1.91 (1.86)	2.73 (2.57)
Free recall: complete pairs	9.67 (4.84)	13.33 (5.75)	11.47 (5.97)	9.80 (4.92)
*Note*. Participants from the crucial sequential probability ratio test (SPRT) subsample and the full sample were excluded if they estimated the alcohol content of their beverage to be less than 1% in the placebo condition and if they reported to have communicated about Phase 1 of the experiment with other participants. *SDs* in parentheses.				

After the application of both exclusion criteria, *N* = 12 participants remained in the subsample, whereas *N* = 62 participants remained in the full sample. The descriptive patterns mirror those obtained without consideration of these exclusion criteria. Similarly, corresponding likelihood ratios and Bayes factors support the conclusion of no clear evidence in favor of H_1_: For Hypothesis 1, LR_12_ = 0.39, LR_62_ = 2.54, BF_10_ = 1.15; for Hypothesis 2, LR_12_ = 0.26, LR_62_ = 1.53, BF_10_ = 0.81.

### Model-Based Results

Hypotheses 3 and 4 referred to the retrieval parameter *r*_f_ and the maintenance parameter *m* of the EMR model, respectively. For the aggregated data, we conducted all analyses in multiTree (Moshagen, 2010). The corresponding preregistered analyses are provided in ESM 6. The baseline EMR model as described in the Data Analysis section did not meet our model fit criterion with α = .05, *G*^2^(11) = 22.00, *p* = .024, AIC = 9,758.71, BIC = 9,824.18. Therefore, we defined a generalized model version with parameters *m*, *u*, and *s* allowed to vary within both conditions between successful and unsuccessful immediate cued recall (*m*_s_ and *m*_u_, *u*_s_ and *u*_u_, and *s*_s_ and *s*_u_, respectively). This generalized model fit the data well, *G*^2^(5) = 5.97, *p* = .309, AIC = 9,754.68, BIC = 9,855.85. A close inspection of this model revealed a significant difference between parameters *u*_s_ and *u*_u_ only within the placebo condition, ∆*G*^2^(1) = 12.55, *p* < .001, but not within the alcohol condition, ∆*G*^2^(1) = 1.06, *p* = .303. Thus, participants in the placebo condition had a significantly higher probability of single word retrieval in the free recall for unsuccessfully stored associations after successful immediate cued recall (*u*_s_ = .21) than after unsuccessful immediate cued recall (*u*_u_ = .10), in contrast to participants in the alcohol condition (*u*_s_ = .16, *u*_u_ = .12).

Accordingly, we defined a new baseline model with the following restrictions: In line with Küpper-Tetzel and Erdfelder (2012), parameters *m*_s_ and *m*_u_ as well as *s*_s_ and *s*_u_ were equated within both experimental conditions, and parameter *r*_c_ was equated across conditions. In contrast, parameter *u* was allowed to vary freely within both conditions between successful (*u*_s_) and unsuccessful (*u*_u_) immediate cued recall. This redefined model yielded a good fit to the data, *G*^2^(9) = 7.43, *p* = .592, AIC = 9,748.14, BIC = 9,825.51. As this model version is more parsimonious than the generalized model version, we decided to use it as our baseline model for the hypothesis tests.

This baseline model was additionally fitted to the individual data in the form of a Bayesian hierarchical model. For this purpose, the model was estimated for both conditions separately, using the latent trait framework of Klauer (2010) as implemented in TreeBUGS (Heck et al., 2018). In this Bayesian framework, convergence of parameter estimates can be evaluated by means of the potential scale reduction factor *R* (Gelman & Rubin, 1992). Good convergence was obtained for all parameters in the alcohol condition, $\hat{R}$ ≤ 1.007, and also in the placebo condition, $\hat{R}$ ≤ 1.003. The model fit was evaluated using the goodness-of-fit statistics *T*_1_ and *T*_2_ (Klauer, 2010). Good model fit was obtained for either condition, as indicated by posterior predictive *p* values of *p*_1_ = .288 (*p*_2_ = .300) in the alcohol condition and *p*_1_ = .565 (*p*_2_ = .420) in the placebo condition. In Bayesian hierarchical MPT modeling, statistical reliability of parameter differences is typically assessed by checking (a) whether the 95% Bayesian credibility interval (BCI) of the posterior distribution of the difference estimate does not include zero (two-tailed) or (b) whether the Bayesian *p* value, that is, the proportion of the posterior distribution of the difference estimate below zero is smaller than the chosen significance level of α = .05 (one-tailed).

After excluding all participants who underperformed (<30% correct) or overperformed severely (>80% correct) in the immediate cued recall, a subsample of *N* = 71 participants remained for the preregistered model-based analysis (31 in the alcohol condition, 40 in the placebo condition). The parameter estimates for both the aggregated and the individual data are presented in Table 4.

**Table 4:** Parameter estimates from the main multinomial processing tree (MPT) analysis

Parameter	Alcohol condition				Placebo condition			
	Aggregated data		Individual data		Aggregated data		Individual data	
	*MLE*	95% CI	*M*	95% BCI	*MLE*	95% CI	*M*	95% BCI
*e*	.58	[.55, .61]	.59	[.53, .64]	.60	[.57, .62]	.60	[.55, .65]
*m*	.92	[.90, .95]	.93	[.90, .96]	.89	[.86, .91]	.90	[.86, .93]
*r*_c_	.98	[.97, .98]	.98	[.97, .99]	.98	[.97, .98]	.98	[.97, .99]
*r*_f_	.50	[.46, .54]	.51	[.45, .56]	.44	[.41, .47]	.43	[.39, .48]
*s*	.05	[.03, .07]	.05	[.03, .07]	.11	[.09, .13]	.11	[.09, .14]
*u*_s_	.16	[.08, .24]	.18	[.07, .34]	.22	[.15, .28]	.20	[.12, .28]
*u*_u_	.12	[.10, .14]	.11	[.08, .15]	.10	[.09, .12]	.11	[.09, .13]
*Note*. For the aggregated data, the encoding-maintenance-retrieval (EMR) model was fitted using the multiTree software (Moshagen, 2010). Maximum likelihood parameter estimates (*MLE*) are presented alongside the corresponding 95% confidence interval (CI). Parameter *r*_c_ was equated across conditions. For the individual data, the model was fitted for both conditions separately using the R package TreeBUGS (Heck et al., 2018). Posterior means (*M*) are presented alongside the corresponding 95% Bayesian credibility intervals (BCI).								

As expected, the probability of associative encoding (parameter *e*) did not differ significantly between conditions, neither for the aggregated data, ∆*G*^2^(1) = 0.55, *p* = .460, nor for the individual data, 95% BCI = [−.09, .06]. Additionally, as expected, parameter *r*_c_ was estimated to be very close to 1 in both modeling approaches.

Based on the interference hypothesis, we predicted a significantly higher probability of associative retrieval in free recall (parameter *r*_f_) for participants in the alcohol condition (Hypothesis 3). Parameter estimates of both modeling approaches were in line with this prediction, and this descriptive effect did reach statistical significance (one-tailed) both for the aggregated data, $z=\sqrt{∆G^{2}\left(1\right)}=2.35,\;p=.009$, and for the individual data, Bayesian *p* = .023.

Based on the consolidation hypothesis, we hypothesized the probability of associative maintenance (parameter *m*) to be significantly higher for participants in the alcohol condition (Hypothesis 4). Again, parameter estimates of both modeling approaches were descriptively in line with our prediction. However, this descriptive effect was statistically significant (one-tailed) only for the aggregated data, $z=\sqrt{∆G^{2}\left(1\right)}=2.33,\;p=.010,$ but not for the individual data, Bayesian *p* = .104.

Overall, the results of our preregistered MPT analysis were in line with Hypothesis 3, but did not yield clear evidence in favor of Hypothesis 4. Additionally, the descriptive effect for parameter *m* was rather small, irrespective of the modeling approach. To further clarify the robustness of this conclusion, we conducted an exploratory MPT multiverse analysis. In this analysis, we not only included our preregistered exclusion criteria of immediate cued recall performance, alcohol content estimation in the placebo condition, and communication during the retention interval (see the “Data Analysis” section), but additionally considered the influence of failure not to drink alcohol within 48 h prior to the experiment and active word pair rehearsal during the retention interval. Thus, our multiverse analysis had a 2 (modeling approach: aggregated vs. individual data) × 2 (model-related exclusion criterion present vs. absent: under- or overperforming in the immediate cued recall) × 2 (at least one procedure-related exclusion criterion present vs. absent: drinking alcohol within 48 h prior to the experiment, estimating the alcohol content to be below 1% in the placebo condition, communicating about the alcohol vs. placebo administration and/or the learning phase, actively rehearsing word pairs during the retention interval) design.

The first two cells of this multiverse design contained our preregistered analyses reported above, where both modeling approaches were applied to the data of all participants who did not meet the model-related exclusion criterion, whereas procedure-related exclusion criteria were not considered. The results of the remaining cells are reported in ESM 7. For both the aggregated and the individual data, the model fit the data well in all remaining cells, thereby allowing for an interpretation of the respective parameter estimates. Overall, our conclusions for both Hypotheses 3 and 4 were confirmed: Parameter *r*_f_ differed reliably between conditions in all cells for the aggregated data (all *p* ≤ .034) and in all cells except one (Bayesian *p* = .051) for the individual data (both other Bayesian *p* ≤ .016). In contrast, parameter *m* differed reliably in only one additional cell (*p* = .010) for the aggregated data (both other *p* ≥ .090) and in no cell for the individual data (all Bayesian *p* ≥ .132).

## Discussion

In the present study, we attempted to replicate the counterintuitive phenomenon of alcohol-induced retrograde facilitation. Using a retention interval of 7 hrs, we followed the basic procedure introduced by Parker et al. (1981) in their original study. Thus, we avoided possible confounds of interpolated sleep and other memory-relevant activities and thereby put the original observation of the effect of interest to a critical test. Moreover, we used a sequential testing procedure (i.e., the SPRT *t* test) to increase the efficiency of data collection and applied the encoding-maintenance-retrieval MPT model to allow for an assessment of the underlying mechanisms. Finally, we decided to conduct and report our study in a registered report format to make the severity of our hypothesis tests transparent (Lakens, 2019).

Contrary to our predictions, we found that drinking alcohol immediately after learning did not significantly increase performance on a delayed subsequent memory test. In line with this finding, there was no clear evidence for a latent maintenance benefit in the alcohol condition, neither in our preregistered MPT analysis nor in an exploratory MPT multiverse analysis. Importantly, however, these MPT analyses revealed a reliable and robust retrieval benefit in the alcohol condition. Thus, although we failed to replicate the original finding by Parker et al. (1981) using conventional measures of memory performance (i.e., cued and free recall), we did find clear-cut evidence in favor of an underlying retrieval benefit (i.e., an effect on EMR parameter *r*_f_).

When evaluating our failure to successfully replicate the results by Parker et al. (1981) on the manifest memory measures of cued and free recall, several methodological differences between the original study and our replication study need to be considered. Most importantly, the results from our MPT analyses suggest that observing a strong and significant retrograde facilitation effect in behavioral measures might require a study design that maximizes the latent contributions of retrieval processes to final memory performance, while minimizing encoding and maintenance contributions. In this respect, it is important to note that Parker et al. (1981) presented their participants with pictures, while we used word pairs as learning material. Crucially, the memory-system dependent forgetting hypothesis (Hardt et al., 2013; Kuhlmann et al., 2021) suggests that hippocampus-dependent memory traces should be less susceptible to retroactive interference than extra-hippocampally represented memories. Thus, the contribution of increased retrievability as a result of reduced retroactive interference might be less pronounced when using paired associates such as word pairs as learning material. Indeed, most other studies reported in the literature used lists of single items instead of paired associates as learning material to demonstrate retrograde facilitation by alcohol (e.g., novel words, Carlyle et al., 2017). On the other hand, Parker et al. (1981) found a significant retrograde facilitation effect on recognition performance, although recognition performance should not or only minimally rely on the retrievability of information from memory.

In contrast to our replication study and the original Parker et al. (1981) study, most studies in the literature used a retention interval between alcohol versus placebo administration and final memory test that was considerably longer than 7 h, ranging between 16 and 48 h (Bruce & Pihl, 1997; Carlyle et al., 2017; Lamberty et al., 1990; Mueller et al., 1983; Parker et al., 1980, study 2; Weafer et al., 2016a, 2016b). As a consequence, these studies could not control for memory-relevant behaviors during the retention interval and necessarily included at least one night of sleep. Against the backdrop of our replication failure, the possibility that the strength of the retrograde facilitation effect was overestimated in these studies due to an effect of acute alcohol intoxication on the sleep architecture – and, thereby, on sleep-induced memory consolidation, potentially benefitting both maintenance and retrieval processes – cannot be dismissed.

Certain limitations of our current study should also be recognized. First, in line with common conventions (cf. Cohen, 1988), we realized a (preregistered) statistical power of 1 − β = .80 and a minimum effect of interest of *d* = .50 for our SPRT *t* tests of Hypotheses 1 and 2. Thus, compared to false-positive findings (probability α = .05), the probability of false-negative findings (β = .20) was relatively high in our study, especially if the true *d* happened to be less than .50. However, a higher statistical power (e.g., 1 − β = .95) for smaller effect sizes (e.g., *d* = .20) would have increased the expected sample size considerably. Thus, we believe to have found a reasonable compromise between sufficient statistical power on the one hand and feasibility of a very resource-intensive study procedure on the other hand.

Second, both SPRT *t* tests terminated rather quickly such that a decision for both Hypotheses 1 and 2 could be made after only *N* = 18 subjects had participated in the study. This outcome nicely highlights the superiority of sequential probability ratio tests in terms of efficiency (cf. Erdfelder & Schnuerch, 2021; Schnuerch & Erdfelder, 2020; Schnuerch et al., 2022). However, in our specific case, the second stopping criterion required for the MPT analyses (*n* ≥ 30 in both conditions after exclusion of participants under- or overperforming in the immediate cued recall) enforced continuation of data collection until a total of *N* = 93 subjects had participated. Thus, our statistical decisions regarding Hypotheses 1 and 2 were based on a fraction of only 19% of the full sample. Although this procedure is statistically valid (i.e., it satisfies the pre-registered statistical error probabilities α and β) and empirically in line with Bayesian *t* test outcomes, we understand that basing hypothesis tests on such a small percentage of the full sample might seem unsatisfactory. A conceivable alternative to our preregistered strategy would have been to start the SPRT *t* tests only when the MPT stopping criterion had already been met. However, although avoiding the aforementioned problem, the efficiency of the data collection process would have suffered from such an approach. In our view, this shows that best practice recommendations of how to optimally combine sequential testing procedures such as the SPRT *t* test with additional stopping rules are needed.

Third, measured peak BACs in the alcohol condition were somewhat lower than anticipated. While the alcohol doses of 0.51 g/kg for women and 0.59 g/kg for men were expected to result in peak BACs of about 0.60‰, the observed mean peak BAC was *M* = 0.43‰. Two factors might be considered to explain this discrepancy. First, peak BACs were most likely reached before or after our measurements for almost all participants, such that our measures underestimate the true peak BACs. Second, alcohol administration was based on the self-reported body weights of participants. It seems reasonable to assume that participants either underestimated and/or knowingly understated their true body weight due to reasons of social desirability. Crucially, the mean peak BAC measured in our study was still higher than in the medium (0.5 ml/kg) alcohol condition of Parker et al. (1981; 0.34‰) for which these authors reported a significant retrograde facilitation effect.

Despite these limitations, our results clearly cast doubt on the reliability, strength, and generalizability of the retrograde facilitation effect in behavioral memory measures. Importantly, the effect is viewed by some authors as a central piece of evidence for the theoretical claim that memory consolidation processes are not limited to periods of sleep, but rather occur whenever retroactive interference is reduced, such as during acute alcohol intoxication (Mednick et al., 2011; Wixted, 2004, 2010). Our lack of evidence for alcohol effects on the probability of maintaining paired associates in memory suggests that such a theoretical model of episodic memory should be treated with caution. Instead, we found that postencoding alcohol administration had a beneficial effect on the probability of retrieving word pairs from memory during free recall. This result is in line with the interference hypothesis, which suggests that retrograde facilitation emerges as a direct consequence of reduced retroactive interference. As such, our finding is more in line with theoretical models that attribute episodic memory improvements (e.g., sleep-induced retrograde facilitation; Rasch & Born, 2013) to retrieval benefits resulting from increased temporal distinctiveness of memory traces (Brown et al., 2007; Ecker et al., 2015).

In sum, our mixed pattern of results best resonates with the idea that alcohol-induced retrograde facilitation really exists but is limited to a retrieval benefit caused by retroactive interference reduction. This idea nicely accounts not only for our confirmation of Hypothesis 3 but also for our failures to confirm Hypotheses 1 and 4 (as both hypotheses refer to memory measures more sensitive to storage than to retrieval from memory). From this theoretical perspective, the only inconsistent outcome is our rejection of Hypothesis 2 concerning free recall. A possible explanation is that overall free recall performance is a less pure measure of retrieval capacity than EMR parameter *r*_f_, which specifically captures retrieval of stored word pairs in free recall.

In terms of future research, more replication studies are needed to specify the exact conditions under which alcohol-induced retrograde facilitation can or cannot be observed. These studies should explicitly consider the role of the learning material (item vs. associative memory) and of sleep and other memory-relevant activities during the retention interval. This implies that more studies are needed that adopt the basic procedure used by Parker et al. (1981) to effectively control behaviors of participants during the retention interval. To make such resource-intensive studies feasible, they should be designed to amplify underlying retrieval differences between conditions (see above), and methodological and statistical innovations such as sequential testing procedures should be embraced. Additionally, as shown by the results of our study, the adoption of MPT modeling for hypothesis generation and testing can be expected to extend possible insights beyond the scope of more conventional analysis strategies. An a priori power analysis using multiTree (Moshagen, 2010) based on the parameter estimates from our pre-registered MPT analysis suggests a minimum total sample size of *N* = 66 across conditions (with 40 word pairs per participant) for future EMR studies that test alcohol versus placebo differences in maintenance and/or retrieval probabilities (condition difference in *m* or *r*_f_ under H_1_ = .10, α = .05, 1 − β = .95).

Alcohol-induced retrograde facilitation is a fascinating psychological phenomenon with potentially far-reaching theoretical and practical implications. However, our results show that more research is clearly needed to provide a solid empirical basis for further discussions of such implications. We hope that future research will build on the methodological and statistical innovations applied in this study to arrive at a deeper understanding of the moderators and mediators of retrograde facilitation by alcohol.

## Electronic Supplementary Material

The electronic supplementary material is available with the online version of the article at https://doi.org/10.1027/1618-3169/a000569

**ESM 1.** This text file provides an illustration of the EMR model and the corresponding model equations.
**ESM 2.** This text file details the calculation of alcohol doses as a function of gender and body weight.
**ESM 3.** This text file provides a complete overview of the learning material.
**ESM 4.** This R script contains all analysis steps to obtain the reported results (except MPT results for aggregated data).
**ESM 5**. This text file depicts the developments of the log-likelihood ratios of both SPRT *t* test.
**ESM 6**. This multiTree file contains all analysis steps to obtain the reported MPT results for aggregated data.
**ESM 7.** This text file contains the results of the MPT multiverse analysis.

## Footnotes

^1^
Due to an initial error in calculating alcohol doses, the doses administered in this study were slightly lower than preregistered for the first *n*_1_ = 28 participants (*N*_total_ = 93), i.e., approximately 0.44 g/kg for women and 0.51 g/kg for men. For the remaining *n*_2_ = 65 participants, the preregistered doses were administered. Crucially, this difference in doses did not result in a relevant difference in peak BAC measurements in the alcohol condition, *M*_1_ = 0.42‰ (*SD* = 0.08), *M*_2_ = 0.44‰ (*SD* = 0.08), *t*(44) = 0.60, *p* = .275, Cohen’s *d* = 0.19. Moreover, the lower-than-preregistered doses still lie above the medium dose of 0.40 g/kg in Parker et al. (1981), which was sufficient to yield a substantial retrograde facilitation effect.

^2^
Due to technical problems during the experiment, BAC measurements are missing for three participants in the alcohol condition 90 min after the end of the alcohol administration and immediately before the final recall tests.

## Notes

**Publication Ethics:** The research protocol was approved by the ethics committee of the University of Mannheim.

**Open Data:** The Stage-1 manuscript of this registered report (https://doi.org/10.17605/OSF.IO/DK8FJ; Quevedo Pütter et al., 2020), the materials (https://doi.org/10.17605/OSF.IO/8E9PW), and the data (https://doi.org/10.17605/OSF.IO/2K4J7) are publicly available on the Open Science Framework (OSF). Additional information needed to reproduce all reported results is provided in the electronic supplemental material (ESM).
