# Supplementary material for: Alcohol-Induced Retrograde Facilitation? Mixed Evidence in a Preregistered Replication and Encoding-Maintenance-Retrieval Analysis
Source: Exp Psychol. 2023 Feb 21;69(6):335–50. doi: 10.1027/1618-3169/a000569 (PMC10388238; doi:10.1027/1618-3169/a000569)
Supplement: Supplementary file 1 [file zea_69_6_335_esm1.pdf]

https://doi.org/10.1027/1618-3169/a000569

Electronic Supplemental Material (ESM) 1

accompanying the manuscript

Alcohol-induced retrograde facilitation? Mixed evidence in a preregistered replication and encoding-maintenance-retrieval analysis

Figure S1

Illustration of the encoding-maintenance-retrieval (EMR) model

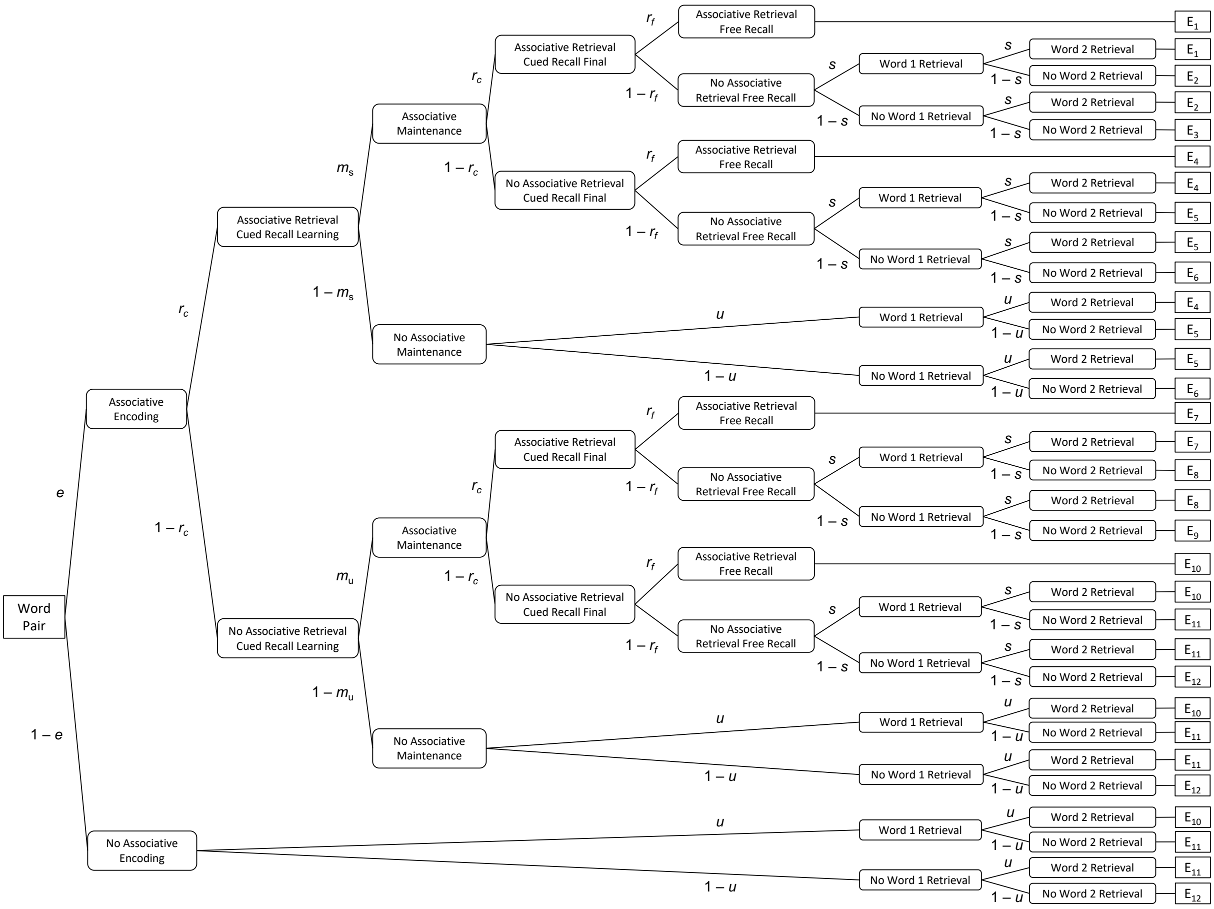

Note. Illustration of the encoding-maintenance-retrieval (EMR) multinomial processing tree (MPT) model (Küpper-Tetzel & Erdfelder, 2012). Each possible sequence of encoding, maintenance, and retrieval processes terminates in one of the observable event categories E_1 to E_{12}. The probability of a branch is just the product of parameters along that branch, and the probability of a category equals the sum of the branch probabilities corresponding to this category.

Applying these rules to the model, the following model equations are obtained:

$p(\mathrm{E}_1) = e\, r_\mathrm{c}^2\, m_\mathrm{s} \left[r_\mathrm{f} + (1 - r_\mathrm{f})\, s^2\right]$

$p(\mathrm{E}_2) = 2\, e\, r_\mathrm{c}^2\, m_\mathrm{s}\, (1 - r_\mathrm{f})\, s\, (1 - s)$

$p(\mathrm{E}_3) = e\, r_\mathrm{c}^2\, m_\mathrm{s}\, (1 - r_\mathrm{f})(1 - s)^2$

$p(\mathrm{E}_4) = e\, r_\mathrm{c} \left[m_\mathrm{s}\, (1 - r_\mathrm{c})\left(r_\mathrm{f} + (1 - r_\mathrm{f})\, s^2\right) + (1 - m_\mathrm{s})\, u^2\right]$

$p(\mathrm{E}_5) = 2\, e\, r_\mathrm{c}\, [m_\mathrm{s}\, (1 - r_\mathrm{c})(1 - r_\mathrm{f})\, s\, (1 - s) + (1 - m_\mathrm{s})\, u\, (1 - u)]$

$p(\mathrm{E}_6) = e\, r_\mathrm{c} \left[m_\mathrm{s}\, (1 - r_\mathrm{c})(1 - r_\mathrm{f})(1 - s)^2 + (1 - m_\mathrm{s})(1 - u)^2\right]$

$p(\mathrm{E}_7) = e\, (1 - r_\mathrm{c})\, r_\mathrm{c}\, m_\mathrm{u} \left[r_\mathrm{f} + (1 - r_\mathrm{f})\, s^2\right]$

$p(\mathrm{E}_8) = 2\, e\, (1 - r_\mathrm{c})\, r_\mathrm{c}\, m_\mathrm{u}\, (1 - r_\mathrm{f})\, s\, (1 - s)$

$p(\mathrm{E}_9) = e\, (1 - r_\mathrm{c})\, m_\mathrm{u}\, r_\mathrm{c}\, (1 - r_\mathrm{f})(1 - s)^2$

$p(\mathrm{E}_{10}) = e\, (1 - r_\mathrm{c}) \left[m_\mathrm{u}\, (1 - r_\mathrm{c})\left(r_\mathrm{f} + (1 - r_\mathrm{f})\, s^2\right) + (1 - m_\mathrm{u})\, u^2\right] + (1 - e)\, u^2$

$p(\mathrm{E}_{11}) = 2\left[e\, (1 - r_\mathrm{c})^2\, m_\mathrm{u}\, (1 - r_\mathrm{f})\, s\, (1 - s) + e\, (1 - r_\mathrm{c})(1 - m_\mathrm{u})\, u\, (1 - u) + (1 - e)\, u\, (1 - u)\right]$

$p(\mathrm{E}_{12}) = e\, (1 - r_\mathrm{c}) \left[m_\mathrm{u}\, (1 - r_\mathrm{c})(1 - r_\mathrm{f})(1 - s)^2 + (1 - m_\mathrm{u})(1 - u)^2\right] + (1 - e)(1 - u)^2$
