# Supplementary material for: Alcohol-Induced Retrograde Facilitation? Mixed Evidence in a Preregistered Replication and Encoding-Maintenance-Retrieval Analysis
Source: Exp Psychol. 2023 Feb 21;69(6):335–50. doi: 10.1027/1618-3169/a000569 (PMC10388238; doi:10.1027/1618-3169/a000569)
Supplement: Supplementary file 2 [file zea_69_6_335_esm2.pdf]

https://doi.org/10.1027/1618-3169/a000569

Electronic Supplemental Material (ESM) 2

accompanying the manuscript

Alcohol-induced retrograde facilitation? Mixed evidence in a preregistered replication and encoding-maintenance-retrieval analysis

The Widmark formula (Widmark, 1932, as cited in Thierauf et al., 2013) allows for estimation of blood alcohol concentrations (BAC's) as a function of alcohol consumed, gender, and body weight. The equation reads as follows:

$$BAC = \frac{A}{r\,W},$$

with *A* = mass of consumed alcohol in g and *W* = body weight in kg. *r* represents the so-called Widmark factor which takes into account the differences in alcohol tolerance between women and men. In German courts, a Widmark factor *r* of 0.6 for women and 0.7 for men is commonly assumed (Thierauf et al., 2013).

In addition to these three variables, two other factors need to be considered, namely the resorption deficit and the passage of time since alcohol consumption. The resorption deficit expresses the fact that not all alcohol consumed is absorbed by the body. Commonly, within the calculation of BAC's, a resorption deficit of 20% is used. Concerning the passage of time, elimination rates of alcohol between 0.10 and 0.20‰ can be assumed (Thierauf et al., 2013).

The calculated alcohol doses for women and men in the present study of 0.51 g/kg and 0.59 g/kg, respectively, can be derived from the Widmark formula, following the procedure outlined by Thierauf et al. (2013). For a woman weighting 63 kg, a BAC of 0.60‰ is achieved with an alcohol mass of A_1 = 0.60‰ x (0.60 x 63 kg) = 22.68 g. For this alcohol mass, the resorption deficit needs to be considered, resulting in 22.68 g / 0.8 = 28.35 g of pure alcohol to be consumed. As participants are allowed exactly 30 minutes to consume the whole beverage, the elimination of alcohol during this time interval needs to be taken into account. This is done by assuming an average elimination rate of 0.15‰ per hour, resulting in a reduction in

intoxication of 0.075‰ in 30 minutes. Accordingly, A_2 = 0.075‰ x (0.60 x 63 kg) / 0.8 = 3.54 g of pure alcohol need to be added to the beverage. This results in a total dose of $A = A_1 + A_2$ = 31.89 g pure alcohol or 31.89 g / 63 kg = 0.51 g/kg. For men, the equivalent calculations result in a dose of 0.59 g/kg. Assuming higher or lower body weights results in identical doses for both women and men, as body weight is incorporated in the calculation of *A* and doses are derived by dividing *A* by the body weight.
