# Supplementary material for: Alcohol-Induced Retrograde Facilitation? Mixed Evidence in a Preregistered Replication and Encoding-Maintenance-Retrieval Analysis
Source: Exp Psychol. 2023 Feb 21;69(6):335–50. doi: 10.1027/1618-3169/a000569 (PMC10388238; doi:10.1027/1618-3169/a000569)
Supplement: Supplementary file 3 [file zea_69_6_335_esm3.pdf]

https://doi.org/10.1027/1618-3169/a000569

Electronic Supplemental Material (ESM) 3

accompanying the manuscript

Alcohol-induced retrograde facilitation? Mixed evidence in a preregistered replication and encoding-maintenance-retrieval analysis

Table S1

Word pairs used as learning material

Cue word	Target word	Category
Zuhause	Stricken	Hobby
Tafel	Radio	Kommunikationsmedium
Muschel	Forelle	Fisch
Schaufel	Beton	Baumaterial
Ufer	Tal	Landschaft
Würfel	Puppe	Spielzeug
Bonbon	Butter	Nahrungsmittel
Petersilie	Rotkohl	Gemüseart
Stock	Bleistift	Schreibgerät
Lakritz	Zigarette	Genussmittel
Student	Bäcker	Beruf
Reklame	Wein	Alkoholisches Getränk
Bild	Stuhl	Möbelstück
Haufen	Mücke	Insekt
Papier	Wolle	Stoffart
Waschbrett	Flöte	Musikinstrument
Natur	Katze	Vierbeiner
Kariert	Gelb	Farbe
Schach	Schwimmen	Sportart
Kessel	Teller	Geschirrteil
Spaziergang	Fichte	Baum
Zirkel	Kneifzange	Werkzeug

Table S1 (continued)

Karte	Vorfahrt	Verkehrshinweis
Prellung	Masern	Krankheit
Hut	Kleid	Kleidungsstück
Bewegung	Ferse	Körperteil
Dunkelheit	Regen	Wetterphänomen
Trichter	Backofen	Küchengerät
Rakete	Fahrrad	Fortbewegungsmittel
Boden	Zimmer	Wohnmöglichkeit
Bogen	Reck	Sportgerät
Aufkleber	Lenkrad	Autozubehörteil
Sommer	Nelke	Blume
Fest	Großvater	Verwandter
Slawistik	Mathematik	Wissenschaft
Interesse	Trauer	Gefühlszustand
Alarm	Holz	Brennbares Material
Verbot	Totschlag	Strafbare Handlung
Parmesan	Pfeffer	Gewürz
Gesang	Amsel	Vogel

Note. Word pairs and categories were taken from Hager and Hasselhorn (1994). Low prototypicality of cue words and high prototypicality of target words for the respective category were determined empirically by Hager and Hasselhorn (1994) by asking participants to generate exemplars for given categories or to rate the representativeness of exemplars for categories. In the present study, target words had to be generated from the respective cue word in the cued recall tasks. Categories were never presented.
