# Supplementary material for: Alcohol-Induced Retrograde Facilitation? Mixed Evidence in a Preregistered Replication and Encoding-Maintenance-Retrieval Analysis
Source: Exp Psychol. 2023 Feb 21;69(6):335–50. doi: 10.1027/1618-3169/a000569 (PMC10388238; doi:10.1027/1618-3169/a000569)
Supplement: Supplementary file 5 [file zea_69_6_335_esm5.pdf]

https://doi.org/10.1027/1618-3169/a000569

Electronic Supplemental Material (ESM) 5

accompanying the manuscript

Alcohol-induced retrograde facilitation? Mixed evidence in a preregistered replication and encoding-maintenance-retrieval analysis

Figure S2

Development of the log-likelihood ratio for the sequential probability ratio *t* test (SPRT *t* test) of Hypothesis 1

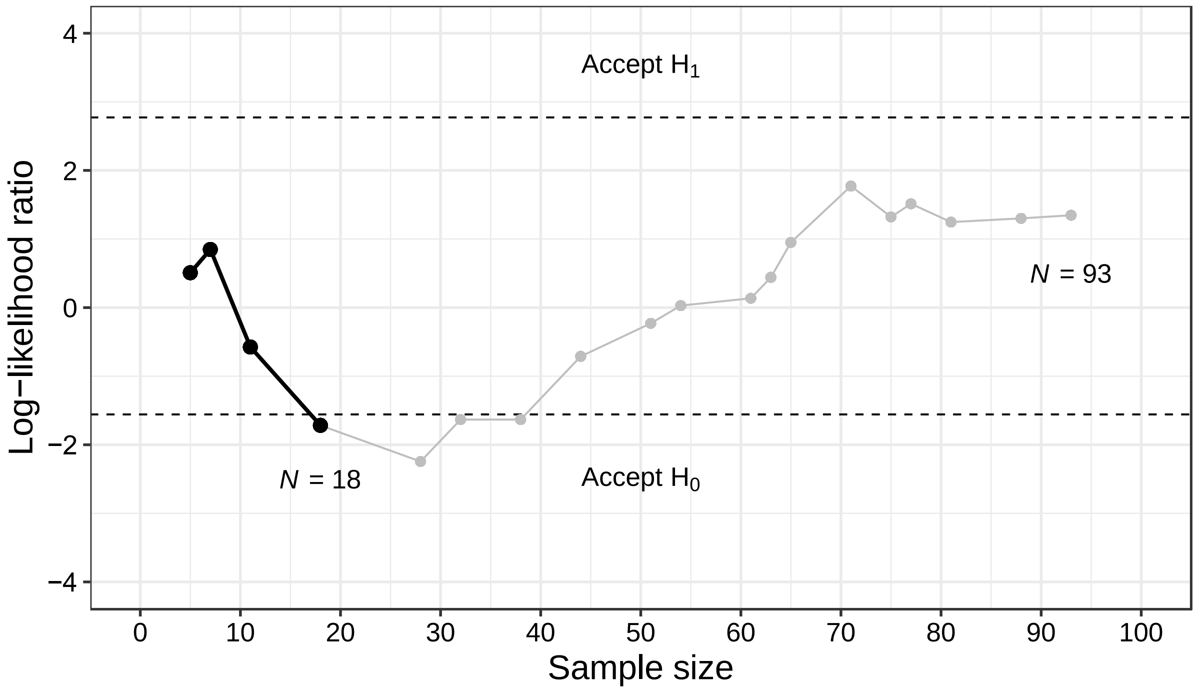

Note. For Hypothesis 1, mean cued recall difference scores (number of correct responses in the immediate cued recall – number of correct responses in the final cued recall) were compared between the alcohol and the placebo condition (one-tailed, α = .05, 1-β = .80, Cohen's *d* = 0.50). Each dot represents the log-likelihood ratio (*LLR*) after the latest group of participants had taken part in the experiment. The lower threshold of -1.56 was reached at *N* = 18, LLR_{18} = -1.72, meaning that H_0 was accepted. Data collection continued until enough participants could be included in the multinomial processing tree (MPT) analysis (*N* = 93). However, only the

SPRT subsample of N = 18 is relevant with respect to the statistical decision regarding Hypothesis 1 (represented by bold dots and lines).

Figure S3

Development of the log-likelihood ratio for the sequential probability ratio *t* test (SPRT *t* test) of Hypothesis 2

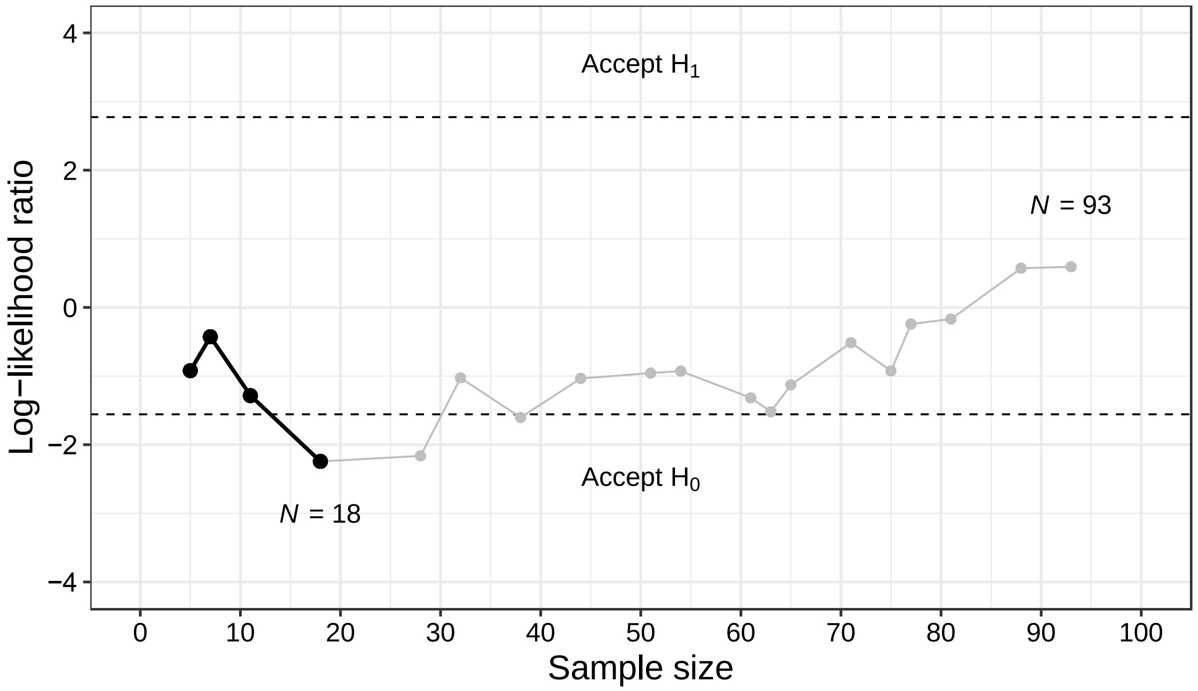

Note. For Hypothesis 2, mean free recall performances (number of complete word pairs reproduced) were compared between the alcohol and the placebo condition (one-tailed, α = .05, 1-β = .80, Cohen's d = 0.50). Each dot represents the log-likelihood ratio (*LLR*) after the latest group of participants had taken part in the experiment. The lower threshold of -1.56 was reached at N = 18, LLR_{18} = -2.24, meaning that H_0 was accepted. Data collection continued until enough participants could be included in the multinomial processing tree (MPT) analysis (N = 93). However, only the SPRT subsample of N = 18 is relevant with respect to the statistical decision regarding Hypothesis 2 (represented by bold dots and lines).
