# Supplementary material for: Alcohol-Induced Retrograde Facilitation? Mixed Evidence in a Preregistered Replication and Encoding-Maintenance-Retrieval Analysis
Source: Exp Psychol. 2023 Feb 21;69(6):335–50. doi: 10.1027/1618-3169/a000569 (PMC10388238; doi:10.1027/1618-3169/a000569)
Supplement: Supplementary file 7 [file zea_69_6_335_esm7.pdf]

https://doi.org/10.1027/1618-3169/a000569

Electronic Supplementary Material (ESM) 7

accompanying the manuscript

Alcohol-induced retrograde facilitation? Mixed evidence in a preregistered replication and encoding-maintenance-retrieval analysis

Table S4

Parameter estimates from the multinomial processing tree (MPT) multiverse analysis without the model-related exclusion criterion and without the procedure-related exclusion criteria

Parameter	Alcohol condition (n = 46)				Placebo condition (n = 47)			
	Aggregated data		Individual data		Aggregated data		Individual data	
	MLE	95% CI	*M*	95% BCI	*MLE*	95% CI	*M*	95% BCI
e	.60	[.58, .63]	.62	[.54, .70]	.61	[.59, .63]	.62	[.56, .68]
m	.93	[.91, .94]	.93	[.90, .96]	.90	[.88, .91]	.90	[.87, .93]
r_c	.98	[.97, .98]	.98	[.97, .99]	.98	[.97, .98]	.98	[.97, .99]
r_f	.51	[.48, .54]	.52	[.47, .56]	.45	[.41, .48]	.44	[.40, .48]
s	.06	[.04, .07]	.06	[.04, .08]	.11	[.09, .13]	.11	[.09, .14]
u_s	.16	[.09, .23]	.16	[.08, .26]	.21	[.15, .27]	.21	[.14, .28]
u_u	.11	[.10, .13]	.12	[.09, .16]	.10	[.09, .12]	.11	[.09, .13]

Note. For the full data (N = 93), the model fitted both the aggregated data, $G^2(9) = 8.06$, p = .528, and the individual data, p_1 = .168 (p_2 = .363) in the alcohol condition, p_1 = .491 (p_2 = .449) in the placebo condition. Maximum likelihood parameter estimates (*MLE*) for the aggregated data are presented alongside the corresponding 95% confidence interval (CI).

Parameter r_c was equated across conditions. Posterior means (*M*) for the individual data are presented alongside the corresponding 95% Bayesian credibility intervals (BCI). For the aggregated data, there was a statistically significant difference (one-tailed) between conditions for parameter m, $z = \sqrt{\Delta G^2(1)} = 2.33, p = .010$, and for parameter r_f, $z = \sqrt{\Delta G^2(1)} = 2.78, p = .003$. For the individual data, there was a statistically meaningful difference (one-tailed) for parameter r_f, Bayesian $p = .006$, but not for parameter m, Bayesian $p = .132$.

Table S5

Parameter estimates from the multinomial processing tree (MPT) multiverse analysis with the model-related exclusion criterion and with the procedure-related exclusion criteria

Parameter	Alcohol condition (n = 19)				Placebo condition (n = 20)			
	Aggregated data		Individual data		Aggregated data		Individual data	
	MLE	95% CI	*M*	95% BCI	*MLE*	95% CI	*M*	95% BCI
e	.59	[.55, .62]	.59	[.52, .67]	.60	[.57, .64]	.60	[.53, .67]
m	.92	[.89, .95]	.92	[.88, .96]	.89	[.86, .92]	.89	[.82, .95]
r_c	.98	[.97, .98]	.97	[.95, .98]	.98	[.97, .98]	.99	[.97, 1.00]
r_f	.50	[.45, .55]	.50	[.42, .58]	.43	[.38, .47]	.42	[.35, .48]
s	.06	[.04, .09]	.07	[.04, .10]	.11	[.08, .14]	.11	[.07, .17]
u_s	.17	[.06, .29]	.18	[.04, .39]	.24	[.15, .34]	.24	[.12, .36]
u_u	.10	[.08, .13]	.10	[.07, .14]	.11	[.08, .13]	.11	[.08, .14]

Note. For this subset of the data (N = 39), the model fitted both the aggregated data, $G^2(9)$ = 12.41, p = .191, and the individual data, p_1 = .429 (p_2 = .573) in the alcohol condition, p_1 = .723 (p_2 = .544) in the placebo condition. Maximum likelihood parameter estimates (*MLE*) for the aggregated data are presented alongside the corresponding 95% confidence interval (CI). Parameter r_c was equated across conditions. Posterior means (M) for the individual data are presented alongside the corresponding 95% Bayesian credibility intervals (BCI). For the aggregated data, there was a statistically significant difference (one-tailed) between conditions for parameter r_f, $z = \sqrt{\Delta G^2(1)} = 2.11, p = .017$, but not for parameter m, $z = \sqrt{\Delta G^2(1)} = 1.34, p = .090$. For the individual data, there was no statistically meaningful

difference (one-tailed) for parameter m, Bayesian p = .221, or for parameter r_f, Bayesian p = .051.

Table S6

Parameter estimates from the multinomial processing tree (MPT) multiverse analysis without the model-related exclusion criterion and with the procedure-related exclusion criteria

Parameter	Alcohol condition (n = 30)				Placebo condition (n = 25)			
	Aggregated data		Individual data		Aggregated data		Individual data	
	MLE	95% CI	*M*	95% BCI	*MLE*	95% CI	*M*	95% BCI
e	.57	[.55, .60]	.59	[.49, .68]	.60	[.57, .63]	.61	[.51, .70]
m	.91	[.89, .93]	.91	[.86, .95]	.90	[.87, .92]	.90	[.85, .95]
r_c	.98	[.97, .98]	.97	[.95, .98]	.98	[.97, .98]	.98	[.97, 1.00]
r_f	.50	[.46, .54]	.52	[.45, .59]	.44	[.40, .48]	.43	[.36, .48]
s	.07	[.05, .09]	.07	[.05, .10]	.11	[.08, .14]	.12	[.08, .16]
u_s	.16	[.08, .24]	.14	[.04, .26]	.22	[.13, .30]	.22	[.11, .33]
u_u	.10	[.08, .12]	.11	[.08, .15]	.10	[.08, .12]	.11	[.08, .14]

Note. For this subset of the data (N = 55), the model fitted both the aggregated data, $G^2(9)$ = 10.13, p = .340, and the individual data, p_1 = .452 (p_2 = .525) in the alcohol condition, p_1 = .632 (p_2 = .371) in the placebo condition. Maximum likelihood parameter estimates (*MLE*) for the aggregated data are presented alongside the corresponding 95% confidence interval (CI). Parameter r_c was equated across conditions. Posterior means (*M*) for the individual data are presented alongside the corresponding 95% Bayesian credibility intervals (BCI). For the aggregated data, there was a statistically significant difference (one-tailed) between conditions for parameter r_f, $z = \sqrt{\Delta G^2(1)} = 1.83, p = .034$, but not for parameter m, $z = \sqrt{\Delta G^2(1)} = 0.67, p = .251$. For the individual data, there was a statistically meaningful

difference (one-tailed) for parameter r_f, Bayesian $p = .016$, but not for parameter m, Bayesian $p = .400$.
